# Quality assurance devices for dynamic conformal radiotherapy

**DOI:** 10.1120/jacmp.v5i1.1955

**Published:** 2004-05-25

**Authors:** Victy Y.W. Wong

**Affiliations:** ^1^ Department of Clinical Oncology Tuen Mun Hospital New Territories Hong Kong China

**Keywords:** quality assurance device, leaf position accuracy, dosimetric verification, IMRT, dynamic conformal arc

## Abstract

Two quality control devices, a light‐field device and a radiation‐field device, have been specially designed to assist the clinical implementation of conformal dynamic arc treatment (CDAT) and intensity‐modulated radiation therapy (IMRT). With these devices, the light field as well as the radiation field projected from the individual beams at any treatment position (i.e., arbitrary gantry angle) can be evaluated. The devices are attached at the front end of the couch and placed at the isocenter of the linear accelerator treatment system (LINAC). The devices are designed to be able to rotate parallel to the gantry head so that the light field and the radiation field projected from a direct beam can be assessed. The aim of this study was to evaluate the geometric precision of the beam placement and the dosimetric accuracy performed in CDAT and IMRT with the aid of these devices. The devices are placed separately from the LINAC during use and provide an independent check on the quality performance of the LINAC in three dimensions. The condition of gantry sagging and any mechanical displacement resulting in field shift can be observed and traced during gantry rotation. Mistakes that occur during the isocenter calibration can lead to significant displacement in the field projection, which would not be revealed with the conventional quality control setting (i.e., gantry 0°). This was demonstrated with the aid of the two quality control devices in the study. The influence of gravitational acceleration in the multi‐leaf collimator (MLC) leaf positioning error, which would consequently lead to inaccurate dose delivery, was investigated. The results of our study show that the existence of a gravitational influence is statistically significant, although the magnitude of the dose inaccuracy is small.

PACS numbers: 87.53.Kn, 87.56.Fc, 87.66.Cd

## I. INTRODUCTION

Over the past decade, the field‐shaping technique has been widely implemented to upgrade conventional radiotherapy to a three‐dimensional conformal radiation treatment. The benefits of dose conformity to the target volume while sparing dose to normal tissues and improving target dose uniformity have been discussed in several publications.^(^ ^1^ ^–^ ^7^ ^)^ The technique is based on adjusting the beam aperture to match the shape of the target at various gantry angles. This was performed initially with a few conformal static beams through the use of a number of custom‐molded blocks made of lead alloy. With the development of the dynamic multi‐leaf collimator (DMLC), the field shape can be dynamically conformed to the target during gantry rotation. Thus, further improvement in dose conformity can be achieved through a series of conformal dynamic arcs. Lately, the field‐shaping technique has been extended into the concept of intensity‐modulated radiation, known as intensity‐modulated radiation therapy (IMRT).

The intensity‐modulated dose distribution is acquired by dividing the treatment fields into a number of segments. Each segment is automatically shaped by the multi‐leaf collimator (MLC) and irradiated as a function of the fraction of the total number of monitor units (MUs). The dose rate and the leaf movement for each segment are calculated by the MLC control system. The accuracy of the MLC leaf position at the isocenter is defined by a tolerance factor that normally ranges from 0.05 cm to 0.5 cm. To achieve a tighter tolerance, in the case of IMRT, if the leaf cannot reach the position in the required time, the treatment time will be increased by the electron gun delay, which results in a reduced dose rate. However, dose variations due to beam instability may be induced by the increase in beam hold‐off incidence^(8)^ In the case of the conformal dynamic arc treatment (CDAT), the change of the field shape is accompanied by the rotation of the gantry, and the speed of the gantry rotation is restricted by the limitation of the dose per arc degree as specified by the system. Therefore, the speed of gantry rotation cannot be adjusted to cater to the leaf positioning accuracy; a certain amount of leaf position error has to be accepted in order to make the treatment deliverable.

Although CDAT and IMRT are characterized by their superior conformal dose distribution compared with conventional modalities, their routine clinical implementation is partially held back by the complexity of their beam verification. Therefore, to ensure that the treatment is delivered accurately, it is essential that an efficient and effective quality assurance program can be applied on a routine basis.

A review of the literature indicates that dose verification is performed during the commissioning of the system to ensure dose accuracy^(^ ^9^ ^–^ ^16^ ^)^ Prior to treatment, a routine quality assurance control program is followed to ensure that the mechanical integrity of the treatment unit is maintained to an established standard. In general, before treatment, the shape of an individual treatment field is checked against the light field or radiation field. For the case of IMRT, it is common to perform a radiation fluence test for individual treatment fields. This test is generally performed at the 0° gantry angle due to the setup expediency. The result of such a test, however, does not necessarily provide the same outcome as if performed at the treatment position. The mechanical displacement of the LINAC system during gantry rotation and the effects of gravitational acceleration on leaf position accuracy are always of concern^(17)^ because these factors can affect dose distribution. Moreover, such a setting cannot be applied to the case of CDAT because the beam shapes are changed during gantry rotation. In this study, two devices are introduced to allow the light and the radiation fields to be tested at any treatment position to confirm whether the mechanical integrity of the treatment system is fulfilled.

## II. MATERIALS AND METHODS

### A. Light‐field test device

**Figure 1:**
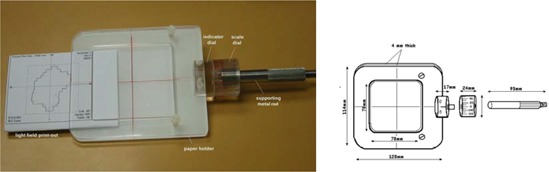
(a) Light‐field device. (b) A schematic diagram of a light‐field device.

The light‐field device is made of Perspex and consists of two concentric dials—a scale dial and an indicator dial—a paper holder, and a supporting metal rod (Fig. 1). The two dials are designed to be able to rotate independently from each other on the same axis. The indicator dial is connected to the scale dial at one end; the paper holder is attached on the other end. An angular scale of 360° in 10° steps and a pointer for indicating the angular rotation are marked along the rims of the scale dial and the indicator dial, respectively. The paper holder is designed to rotate in unison with the indicator dial. The amount of rotation can be traced on the scale dial at the corresponding position as indicated by the pointer of the indicator dial. A center cross is marked on the paper holder, which illustrates the axis of rotation of the system.

The paper holder consists of two pieces of Perspex with dimensions 128 mm×113 mm×4 mm. A light‐field printout generated by the planning system is held between the Perspex sheets during the light‐field test. To avoid the production of light diffraction during the light‐field test, a square opening with dimensions 78 mm×78 mm was made in the middle of the upper sheet. The entire system is supported by a metal rod, which is inserted through the central axis of the scale dial and acts as a supporting pole for rotation.

### B. Radiation‐field test device

**Figure 2:**
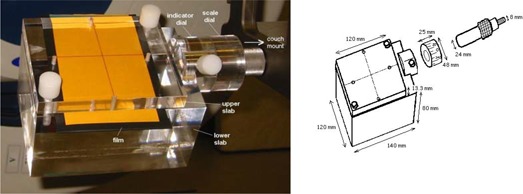
(a) Radiation‐field device. (b) A schematic diagram of a radiation‐field device.

The radiation‐field device (Fig. 2) is very similar in design to the light‐field device. However, instead of the paper holder, a block of Perspex is used. The Perspex block is divided into two slabs. For attenuation of 6 MeV photons, the upper slab is chosen to be 13.3 mm thick, which is equivalent to a water depth of 15 mm. The lower slab has a thickness of 80 mm to eliminate back‐scattered electrons primarily produced by the 6 MeV photons. A film placed between the upper and lower slabs is secured by screwing the two Perspex slabs together. A center cross is marked on the upper slab to indicate the rotational axis of the whole system.

### C. Device calibration

Before using, the device must be calibrated at the horizontal level to define the offset position (0°). Calibration is performed with the device attached to the treatment couch. Instead of securing the device directly onto the treatment couch, a stereotactic couch mount is applied to ease the setup procedure because the couch mount provides for level adjustment. The offset position is found by rotating the paper holder/Perspex block together with the indicator dial to a horizontal level. The horizontal level is confirmed by placing water on the surface of the paper holder/Perspex block along the transverse axis of the treatment couch. When the horizontal level is achieved, the pointer marked on the indicator dial indicates the offset position. The offset position is then denoted with the scale dial at the position marked with 0 division. The horizontal level along the longitudinal axis of the treatment couch is also adjusted with the couch mount. After calibration, the position of the scale dial must be kept constant throughout the whole test. The center of the paper holder/Perspex block is then brought to the position of the isocenter of the treatment system by aligning it with the wall lasers and the ceiling lasers. Before the light‐field or radiation‐field test, the horizontal level of the holder must be checked after the printout/film has been inserted to ensure that no tilting results from the thickness of the printout/film. Direct beam projection is obtained by rotating the paper holder/Perspex block and placing it at the same angle as the gantry. Since the indicator dial is rotated with the paper holder/Perspex block, the angle of rotation is shown on the scale dial at the position pointed by the indicator dial.

### D. Tests for light field and radiation field

The automation precision of the LINAC system applied for the treatment of DCAT and IMRT was evaluated with the aid of the light‐field and radiation‐field devices. The studies were taken on a Varian CLINAC 2100 C/D equipped with an m3™ (BrainLab, AG, Germany) micro‐multileaf collimator (mMLC). The mMLC consists of 26 pairs of tungsten leaves that allow the treatment field to be shaped up to $10\;{\text{cm}}^{2}\times 10\;{\text{cm}}^{2}$. The leaf width ranges from 3.0 mm at the center of the field to 5.5 mm at the periphery. The maximum distance over the center line is 5 cm, and the maximum separation between the leaves is 10 cm. The system is capable of both dynamic MLC (DMLC) and segmental MLC (SMLC) delivery techniques; however, only the DMLC delivery technique is applied in this study. During IMRT, the control system drives the leaves at their maximum speed of 1.5 cm/s in a stepping manner while modulating the dose rate to achieve the desired leaf position. Various dose rates are applied in a range of 100 MU/min to 600 MU/min. The default tolerance factors of 0.25 cm and 0.5 cm are specified by the vendor, which controls the spontaneous MLC leaf position in IMRT and CDAT, respectively. According to the vendor's specifications, leaf position accuracy is also governed by the secondary feedback and the stepping motor of the system, which drive the leaf to the position with an accuracy of 0.1 mm. The test results of the light and radiation fields were compared with the dose plan generated by the planning system (BrainScan v.5.1, BrainLab AG). The studies performed were as follows:

1. The accuracy of the field projection of a dynamic conformal arc at different gantry angles was studied with the aid of the light‐field device. The field shape projected from the gantry was compared at every 10° of the gantry angle with the light‐field printout generated by the planning system.
2. The effect of gravitational acceleration on the leaf position and the dosimetric error during IMRT were evaluated. Two clinical plans with 13 IMRT fields were applied for this study. The maximum dimensions of the target volumes were 27 mm×22 mm×38 mm and 50 mm×50 mm×28 mm. To study the gravitational effect, each field was repeated to irradiate in three settings: setting 1: gantry 0° and collimator rotation 0°; setting 2: gantry 90° and collimator rotation 0°; and setting 3: gantry 90° and collimator rotation 90°. When both the gantry and collimator rotations are at 90°, the direction of the MLC leaf motion is parallel to the effect of gravity. In this case, any gravitational acceleration acting upon the MLC during gantry rotation will result in an increase in the leaf position error. In the case of gravitational influence, the difference in dose distribution between settings 3 and 1 are expected to be significantly higher than that between settings 2 and 1.
  A film was placed between the two Perspex slabs of the radiation‐field device and was irradiated according to the value of the leaf sequence factor. Radiation, in the amount of 100 MU to 148 MU was given. To avoid dose response saturation, the dose response sensitivity of the Kodak Xomat‐V film was tested in a dose range of 0 MU to 250 MU. The film was placed at the isocenter with a 15 mm buildup of water. In the study of each field, each set of three films was developed at the same time and followed by the dose scan with the film scanner (VIDAR VXR 12 plus) to minimize the variations that arose during film processing and scanning. The dose distributions were studied with a film dosimetry system (RIT v.3.13, Radiological Imaging Technology, Inc.). Using the dose distributions obtained at setting 1 as a reference, the differences between dose distributions obtained in settings 2 and 3 were compared.
3. The displacement of the beam projection resulting from the inaccurate calibration of the isocenter was studied with the aid of the radiation device. In the study, the isocenter was inaccurately calibrated by a 1.1 mm shift from the actual position. The beam fluence profile of a single IMRT field was studied at three gantry angles: 0°, 50°, and 90°. The amount of field displacement was assessed by image subtraction. The fluence images obtained at the shifted isocenter were subtracted by that obtained at the correct isocenter at gantry zero.

## III. RESULTS

**Figure 3:**
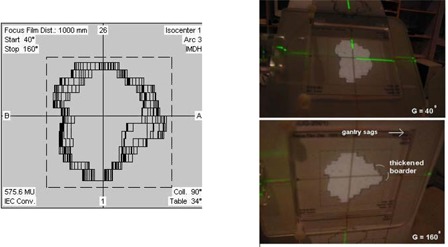
(a) A printout of overlapped light fields of a dynamic conformal arc from 40° to 160° was generated by the planning system. The change of the beam shape can be monitored step‐by‐step with the light‐field device. (b) The light‐field projection at gantry 40° corresponded perfectly to the printout. Gantry sagging was observed by the increasing displacement of the light field from 90° to 160° beam projection, as shown by the thickened field border.

Fig. 3(a) shows a printout of a dynamic conformal arc with the overlapped light fields from 40° to 160° generated by the planning system. The change of the beam shape can be tested step‐by‐step with the light‐field device. Fig. 3(b) shows that the light field projected at the gantry angle of 40° is perfectly matched with the printout. However, gantry sagging was noticed by increasing the displacement of the light field from 90° to 160° of beam projection, as shown by the thickened field border.

The dose response sensitivity of the Kodak Xomat‐V film was studied by plotting the optical density against doses in the range of 0 MU to 250 MU. The result confirms that the dose range (100 MU to 148 MU) applied in our study is well beyond the dose response saturation region (the plateau of the curve).

**Figure 4:**
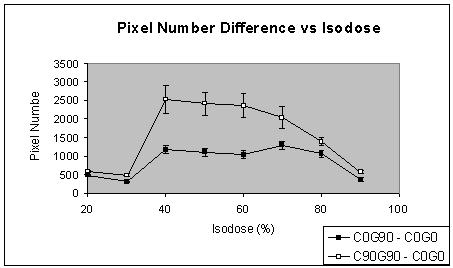
The mean differences in pixel number between setting 3 (gantry 90°, collimator rotation 90°) and setting 1 (gantry 0°, collimator rotation 0°), and between setting 2 (gantry 90°, collimator rotation 0°) and setting 1 of 13 fields, were plotted as a function of isodoses in the range 20% to 90%.

The differences between the dose distributions obtained in setting 2 (gantry=90°, collimator=0°) and setting 3 (gantry=90°, collimator=90°) were studied using the dose distribution obtained in setting 1 (gantry=0°, collimator=0°) as a reference. By using setting 1 as a reference, the variations in pixel‐to‐pixel response, processing, and scanning conditions were accounted for and eliminated. Doses were normalized to the maximum dose of individual films. The dose histogram was studied, and the number of pixels enclosed by the isodoses from 20% to 90% in 10% steps was calculated. Neither extremely low (<20%) nor extremely high doses (>20%) were used for the study due to the substantial errors induced by the insensitive detection in the low‐dose region during film scanning and the limitation in spatial resolution for small area dose. The differences in pixel number between settings 3 and 1 and between settings 2 and 1 as a function of isodoses from 20% to 90% were calculated for all fields. The mean differences in pixel number and the standard deviations of 13 fields were plotted against the isodoses, as shown in Fig. 4. The unit pixel size is 339 μm. Fig. 4 shows a substantial difference, in terms of dose coverage, in settings 3 and 2 using setting 1 as a reference. The mean differences in pixel number between settings 3 and 1 and between settings 2 and 1 as a function of isodoses range from 482 to 2525 and 296 to 1295, respectively. This is equivalent to the area coverage of 0.6 cm^2^ to 2.9 cm^2^ and 0.3 cm^2^ to 1.5 cm^2^, respectively. The difference in the number of pixels between settings 3 and 1 and between settings 2 and 1 were compared in pairs as a function of isodose from 20% to 90% in steps of 10% for all fields. The result of the paired *t*‐test suggested a significant difference (p<0.001) in dose coverage between settings 3 and 2. Our results therefore support that the position that gravitational acceleration influences leaf position error, which subsequently leads to inaccurate dosimetry.

**Figure 5:**
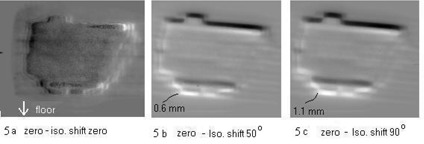
Displacement of beam projection resulting from the inaccurate calibration of the isocenter by a 1.1 mm shift in the vertical direction is demonstrated with an IMRT field projected at angles 0°, 50°, and 90°. No displacement was observed at 0°, as shown in (a), and increasing displacement was noticed as the gantry moved away from 0°. The amount of displacement can be observed on the subtracted radiation images, with the isocenter shift images subtracted by an image without isocenter shift projected at 0°. Displacements in beam projection of 0.6 mm and 1.1 mm in vertical direction were documented at the gantry angles 50° and 90°, as shown in (b) and (c), respectively.

Fig. 5 demonstrates the displacement of the radiation field resulting from the inaccurate calibration of the isocenter. The isocenter was miscalibrated with 1.1 mm shifted vertically away from the actual position. This is shown by an IMRT field projected at gantry angles of 0°, 50°, and 90°; no displacement was observed at 0°. However, increasing displacement was noticed as the gantry moved away from 0°. The amount of displacement was assessed by image subtraction. The image that was taken at the shifted isocenter was subtracted by the image without isocenter shift obtained at 0°. The images were found with a displacement of 0.6 mm and 1.1 mm in the vertical direction as the gantry moved to 50° and 90°, respectively.

## IV. DISCUSSION

The devices introduced in this study provide an independent check on the accuracy of the automation performance of the LINAC and the mMLC system. Since the device is positioned at the isocenter and separated from the LINAC system, it offers an accurate geometrical reference without error induction from the movement of the LINAC system. The current commercially available equipment, such as the stereotactic film holder produced by BrainLab and the portal imager, may be applied for such studies. However, these devices are attached to the gantry and rotated about the same axis as the gantry head. It is difficult to differentiate any geometrical inaccuracy caused by gantry sag, gantry displacement, and inaccurate system settings (e.g., isocenter calibrations, gantry angles calibration). Moreover, due to the weight of the tools themselves, perfect circular rotation along the circumference is impossible because displacement caused by sagging is often experienced when the film holder/portal imager starts moving away from the zero gantry angle.

With the devices introduced here, the above‐mentioned problems have been overcome; hence, the mechanical integrity of the LINAC system can be examined in a precise manner. The accuracy of the speed response of the MLC during DCAT and IMRT is also an important factor to ensure accurate dosage. Whether this will be affected by the orientation of the gantry head due to the effect of gravitational acceleration should be considered. Our study proves that there is a certain influence in leaf position accuracy due to gravitational acceleration. By restricting the tolerance level, one can minimize the effect and further improve dosimetry accuracy; however, it should be compromised with the practical capability of the system. Therefore, it is recommended that the optimal tolerance level be carefully investigated for each treatment system in such a way that it is practically tolerable and conceded with the acceptable dose accuracy. Moreover, with the aid of the radiation device, the dose distribution of an individual IMRT field can be obtained at any treatment position. Any inherent mechanical defects can be revealed by comparing the dose distribution with the fluence profile calculated by the planning system.

## V. CONCLUSIONS

Two quality control devices have been specially designed for light‐field and radiation‐field tests for the treatment of CDAT and IMRT. The devices provide an independent check on the mechanical performance of the LINAC system, which demonstrates geometric accuracy of beam delivery. In addition, dosimetry accuracy can be evaluated. With the devices, mechanical deterioration of the gantry can be traced easily in any gantry position. Moreover, mistakes made during system calibration can lead to significant errors in field placement that generally cannot be observed with the conventional quality control setting (gantry 0°) but can be revealed with the aids of these devices. IMRT and CDAT can be regarded as complex treatments that involve complicated dose calculation and complex processes in dose delivery. The devices suggested here are effective and expedient tools that can be used to ensure that the mechanical integrity of the delivery system is maintained to an established standard.

## ACKNOWLEDGMENTS

The author thanks Hei Yuen Choi for the construction of the devices, Rachel Wong for the illustration, Kristal Lau for preparation of the manuscript, and Martin Szegedi from BrainLab Ltd. for the technical discussion.
